# Nest Architecture Drives Sex-Specific Emergence Success in a Predator Wasp (Hymenoptera, Vespidae, *Discoelius wangi*)

**DOI:** 10.3390/insects16121197

**Published:** 2025-11-25

**Authors:** Xue-Li Xie, Hai-Xia Lu, Michael Orr, Ting-Ting Du, Jing-Ting Chen, Xiao-Yu Shi, Rui Cheng, Qing-Song Zhou, Arong Luo, Chao-Dong Zhu, Peng-Fei Guo

**Affiliations:** 1 College of Pharmacy, Guizhou University of Traditional Chinese Medicine, Guiyang 550025, China; 2 Entomologie, Staatliches Museum für Naturkunde Stuttgart, 70191 Stuttgart, Germany; michael.christopher.orr@gmail.com; 3 Key Laboratory of Animal Biodiversity Conservation and Integrated Pest Management, Beijing 100101, China

**Keywords:** Zethinae wasps, *Discoelius wangi*, intercalary cell, nest diameter, vestibular length, subtropical forest, southwestern China, trap nests

## Abstract

Predatory insects play a crucial role in maintaining ecosystem balance. Among them, members of the subfamily Zethinae, as natural enemies of herbivorous pests, have reproductive success closely linked to nest architecture, as this limits their prey items. We set up trap nests for *Discoelius wangi* Yamane, 1996 in a subtropical forest in southwestern China to investigate the effects of nest architecture parameters (number of intercalary cells, nest diameter, and vestibule length) on the number of brood cells, the quantity of male and female offspring, and emergence rate via generalized linear models and hierarchical partitioning. The results showed that for the number of nest cells, only the number of intercalary cells had a significant positive effect. For the quantity of male and female offspring, nest diameter, and the number of intercalary cells had significant positive effects on female offspring, while males were only significantly positively affected by the number of intercalary cells. For emergence rates, female emergence rate was marginally significantly affected by nest diameter, male emergence rate was marginally significantly affected by the number of intercalary cells, and total emergence rate was significantly influenced by vestibular length and the number of intercalary cells. This study indicates that *D. wangi* can adjust its nest characteristics to achieve precise regulation of reproductive performance. The results not only enhance our understanding of how human activities affect predatory insects in forest ecosystems but also provide a scientific basis for developing effective conservation and utilization strategies.

## Simple Summary

This study contributes to understanding how solitary wasps achieve precise regulation of their reproductive performance by adjusting nest architecture parameters in a differentiated manner. This strategy reflects the refined adaptive mechanisms developed by this species during the long-term evolutionary process. The research findings will provide a theoretical basis for the artificial breeding and ecological conservation of solitary wasp species.

## 1. Introduction

Across forest ecosystems, predatory insects act as a pervasive and stabilizing force that shapes trophic interactions and sustains ecosystem functioning. Predatory insects, in particular, operate as cryptic yet powerful regulators whose top-down influence can propagate through entire multitrophic networks. A recent study has highlighted the pivotal role of predatory insects in maintaining the balance and function of ecosystems [1]. By restricting the population size of herbivorous organisms, they directly reduce the damage to plants, and at the same time promote material cycling and energy flow between trophic levels, thus becoming an indispensable regulatory force in ecosystems. Research has found that tree species diversity enhances insect community diversity (particularly predatory wasps) and reduces herbivorous insect damage through the “natural enemy regulation effect,” thereby indirectly promoting forest productivity [1]. Further research has confirmed that the predation of herbivorous insects by predatory wasps plays a crucial role in maintaining the balance and functioning of ecosystems [2]. Despite their recognized importance, the specific functional contributions of many predatory wasp groups remain inadequately studied. In particular, a deeper understanding of their foraging behavior, prey preferences, and regulatory effects within different habitat types is essential for accurately quantifying their ecological impact and implementing effective conservation strategies. This knowledge gap underscores the necessity of further research focused on these keystone predators.

The subfamily Zethinae is a relatively small but important group within the family Vespidae of the order Hymenoptera [3,4]. Adults of most species in this subfamily are herbivorous, feeding on nectar at flowers, while their larvae are carnivorous, relying primarily on lepidopteran larvae (caterpillars) provisioned by the adults [5]. As such, they play a crucial role as natural enemies in regulating populations of herbivorous pests. Their population dynamics influence the trophic balance of the “plant-herbivorous insect” system [6]. As the core microhabitat for cavity-nesting wasps’ reproductive behavior, nest architecture—characterized by parameters such as diameter, number of intercalary cells, and vestibule length—determines nesting success and offspring survival by regulating microclimate, defending against natural enemies, and optimizing resource allocation [7,8].

The diameter of brood cells may influence the stability of the internal microenvironment and the efficiency of resource allocation within the nest [9]. For example, larger-diameter cells, while providing more space for growth, are more susceptible to external influences due to their larger volume, leading to significant fluctuations in temperature and humidity [7]. Additionally, cell diameter affects the distribution of resources such as pollen and nectar, as well as the range of larval activity [10].

Intercalary cells, as special non-reproductive structures within the nest that appear as “empty cells,” physically separate functional areas such as resource storage and larval development zones, reducing mutual interference and potentially enhancing reducing mortality [11,12]. Variations in their size, shape, and number may further influence larval development progress [13].

The length of the vestibule located before the first brood cell is critical for buffering external disturbances [14]. A longer vestibule acts as a natural barrier, effectively mitigating sudden changes in temperature and humidity, predator or parasite attacks, and weather impacts, thereby creating a secure and stable internal environment for larval development [15]. In contrast, a shorter vestibule offers limited buffering capacity, making it difficult to counteract external changes, which can lead to environmental fluctuations within the nest and threaten larval survival [15]. Although the importance of nest characteristics for larval nesting success and offspring survival is gradually seeing more study, many gaps remain in our understanding of their intricate interconnections and underlying mechanisms.

**Figure 1:**
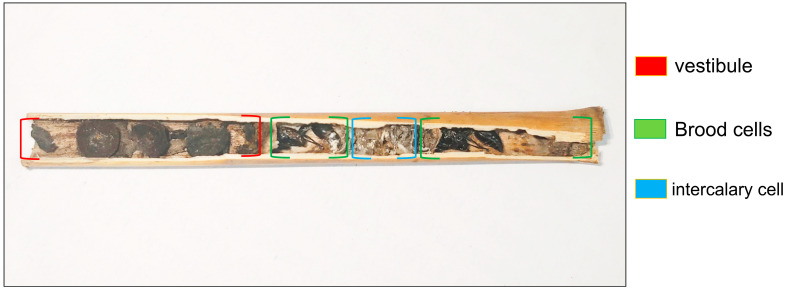
A complete cross-sectional sample of the nest of *Discoelius wangi*, clearly showing the morphological characteristics of the vestibule structure, brood cells, and intercalary cells.

To refine our knowledge of the dynamics of nesting biology, this study focuses on the predatory wasp *Discoelius wangi* Yamane, 1996. By employing trap nests in the subtropical mountain forests of southwestern China, the nesting behavior of this species was monitored across multiple years. Key parameters of nest structure were systematically recorded, including the number of intercalary cells—this parameter refers to the count of all non-reproductive “empty chambers” within the nest—nest diameter (i.e., the inner diameter of the brood cells, which matches the inner diameter of the reed stem) and vestibular length, defined as the distance from the mouth of the reed tube to the opening of the first brood cells (Figure 1). Based on these data, a systematic analysis was carried out around three core research directions: (1) the biological significance of the nesting characteristics (material, layout, sex ratio) of *D. wangi*; (2) differences in the effects of nest architecture parameters on brood cell number and the quantity of male and female offspring; (3) the regulatory mechanisms by which nest architecture affects the emergence rate of male and female offspring (the ratio of the number of emerged males or females to the number of brood cells); and overall emergence success. This study aims to identify the key structural elements underlying the reproductive strategies of *D. wangi*, provide a theoretical basis for the ecological conservation and even artificial breeding of Zethinae species, and enrich the research system on the functional role of nest architecture in solitary Hymenoptera and the mechanisms of insect reproductive adaptation within a multi-trophic framework.

## 2. Materials and Methods

### 2.1. Study Site

This study was conducted in the Chishui Alsophila National Nature Reserve, Guizhou Province (28°20′19″–28°28′40″ N, 105°57′54″–106°7′17″ E). Located in the transition zone from the Guizhou Plateau to the Sichuan Basin, the reserve covers a total area of 133 km^2^, including a 55 km^2^ core zone, a 40 km^2^ buffer zone, and a 38 km^2^ experimental zone. It is one of the key protected areas for subtropical biodiversity in China [16].

### 2.2. Sampling

A standardized trap nest system was used to investigate the regulatory effects of nest architecture on the nest cell number, offspring quantity, and emergence rate of *D. wangi*. The experiment included transects in the Chishui Alsophila National Nature Reserve. A single linear transect, approximately 3 km in length, was established. Consecutive sampling points were placed at 50 m intervals along this transect, ensuring a straight-line distance of 50 m between adjacent points, resulting in a total of 104 sampling points.

At each sampling point, four trap nests (220 mm × 110 mm) made of PVC were installed. In each plot, two posts were installed 7 m apart from each other, each equipped with two trap nests (1.5 m above ground). Each trap nest was filled with 80 ± 10 (SD) standardized reed stems (20 cm in length, 0.3–3 cm in diameter), providing approximately 160 potential nesting cells per trap nest [17].

The trap nests were arranged in an east–west direction. GPS was used to accurately determine the elevation and coordinates of each sampling point. The trap nests were fixed to tree trunks at a height of 1.5 m above the ground using rust-proof wire. Long-acting insect glue was applied to the upper and lower parts of the supporting tree trunks to prevent predation by ants. From 2022 to 2024, trap nests (i.e., tubes with nests built by host cavity-nesting Hymenoptera) were collected monthly from the sampling plots in the reserve and brought back to the laboratory for observation, while replenishing new reeds.

### 2.3. Data Collection and Processing

Nest tubes were processed in the laboratory by first measuring each tube and recording the relevant data. Each tube was then split longitudinally, and detailed observations were recorded, including collection time, the number of brood cells, and the type of provisions. Following data collection, the samples were placed in glass culture tubes with controllable temperature and humidity, and the tube openings were sealed with sterilized absorbent cotton. Following the sealing of the glass tubes, continuous observation was maintained until all adults had emerged (typically 1–3 months post-collection, depending on the developmental cycle of *Discoelius wangi*). For individuals that did not emerge within the same year, the samples were stored under stable room temperature conditions and monitored until eclosion occurred in the following year. For emerged samples, preservation and treatment are carried out after the end of the emergence period; for unemerged samples, preservation and treatment are carried out according to the season and developmental characteristics. During this period, if mold growth or other conditions that can be directly judged as death by the naked eye occur, the samples should be discarded in a timely manner. For samples that are difficult to directly judge, sufficient time is given for them to emerge. Only after confirming that there are no surviving individuals are they discarded. This is to avoid the accumulation of samples, while ensuring that wasps with emergence potential are not mistakenly discarded and that data integrity is maintained.

### 2.4. Statistical Analyses

All statistical analyses were performed using R software (version 4.4.3).

Considering the volume of data in this experiment, we employed generalized linear models (GLM) to analyze the effects of nest parameters (nest diameter, vestibule length, and number of intercalary cells) of *D. wangi* on the number of brood cells, the number of offspring, and the offspring emergence rate. To improve variance homogeneity, we used the glm.nb function in the “MASS” package to fit a negative binomial distribution model for analyzing the number of brood cells and the number of offspring. Meanwhile, we performed log-transformation and standardization (mean = 0, standard deviation = 1) on nest diameter, vestibule length, and the number of intercalary cells.

Since the emergence rates of females, males, and the total emergence rate are all proportional data, a binomial distribution model was used for analysis. Subsequently, the stepwise forward method was employed to select the optimal model. Finally, we used hierarchical partitioning to assess the relative importance of the three key variables—nest diameter, number of intercalary cells, and vestibule length—and employed the “glmm.hp” package to calculate the variance components (R^2^) of these three variables in the GLMMs, as well as the effect sizes of individual predictor variables.

## 3. Results

### 3.1. Nest-Building Status of Discoelius wangi in Trap Nests

A total of 96 nest tubes of *D. wangi* were obtained from an overall collection of 3265 occupied reeds, representing an occupancy rate of 2.94% among all trap-nesting hymenopteran nests recorded in the study system. Overall, 209 offspring of *D. wangi* emerged, including 90 females and 119 males. This species uses fragmented leaves to seal nests and construct brood cell walls; the brood cells are arranged linearly, with an average of 2.96 ± 0.16 cells per nest. The number of intercalary cells per nest ranged from 0 to 4, with an average of 1.49 ± 0.12. The diameter of the brood cells ranged from 4.10 mm to 13.47 mm, with an average of 8.23 ± 0.22 mm. The length of the nest vestibule ranged from 0 mm to 85.91 mm, with an average of 22.50 ± 1.97 mm. The offspring fed on larvae of multiple Lepidoptera species.

### 3.2. Impacts of Nest Architecture on the Number of Brood Cells of D. wangi

**Figure 2:**
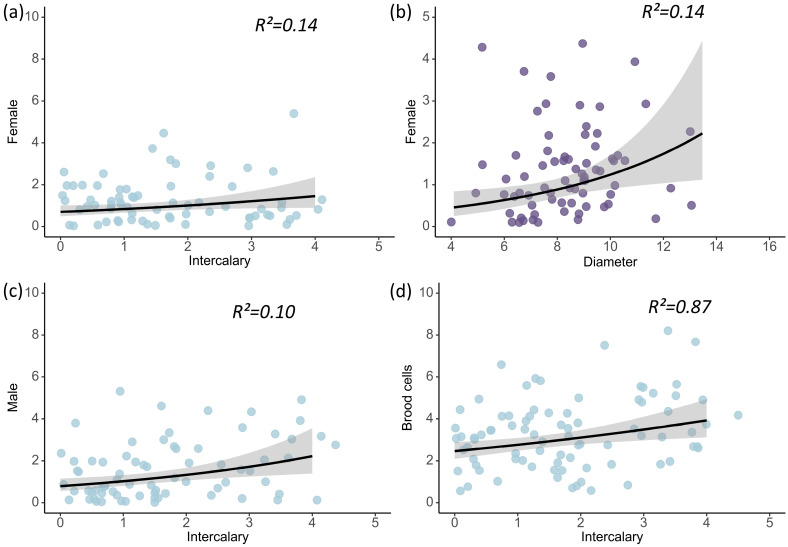
Relationship between the female number of *D. wangi* and number of intercalary cells (**a**), and nest diameters (**b**). Relationship between the male number of *D. wangi* and nest diameters (**c**). Relationship between the brood cells of *D. wangi* and number of intercalary cells (**d**). The gray area represents the 95% confidence interval.

In the model, the number of intercalary cells had a significant positive relationship with the total number of brood cells (Table 1, Figure 2d; Estimate = 0.141 ± 0.061 SE, Z = 2.324, *p* = 0.020 significant averaged model parameters are given in Table 1; full parameters of averaged models can be found in electronic Supplementary Material, Table S1, the hierarchical segmentation map of the full model is shown in Figure S2 of the Supplementary Materials); in contrast, neither the vestibule length (*p* = 0.287) nor the nest diameter (*p* = 0.550) showed a significant relationship.

### 3.3. Effects of Nest Architecture on the Offspring Quantity of D. wangi

**Figure 3:**
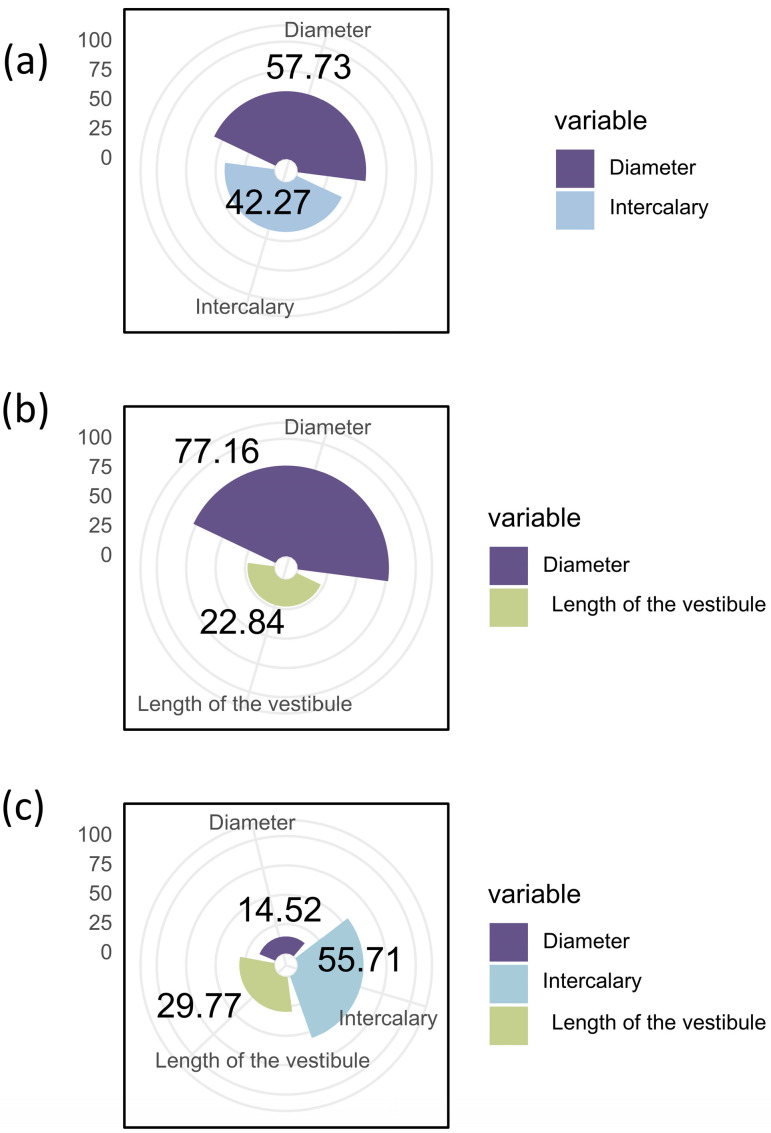
Relative contributions of nest diameter and intercalary cells to the female number of *D. wangi* (**a**). Relative contributions of nest diameter and length of the vestibule to the female emergence rate of *D. wangi*. (**b**) Relative contributions of nest diameter, intercalary cells, and length of the vestibule to the total emergence rate of *D. wangi* (**c**).

In the model, both nest diameter (Table 1, Figure 2b; Estimate = 0.275 ± 0.116 SE, Z = 2.373, *p* = 0.018) and the number of intercalary cells (Table 1, Figure 2a; Estimate = 0.233 ± 0.117 SE, Z = 1.999 *p* = 0.046) were significantly positively related to the number of females; the vestibule length showed no significant relationship (*p* = 0.534). The model showed that the R^2^ is 14.43% of the variation in the number of females. Specifically, diameter explained 57.73% of the variation in the number of females, and the number of intercalary cells explained 42.27% (Figure 3a).

Only the number of intercalary cells had a significant positive relationship with the number of males (Table 1, Figure 2c; Estimate = 0.291 ± 0.114 SE, Z = 2.552, *p* = 0.011); neither the nest diameter (*p* = 0.881) nor the vestibule length (*p* = 0.962) exhibited a significant relationship.

### 3.4. Effects of Nest Architecture on the Offspring Emergence Rate of Discoelius wangi

**Figure 4:**
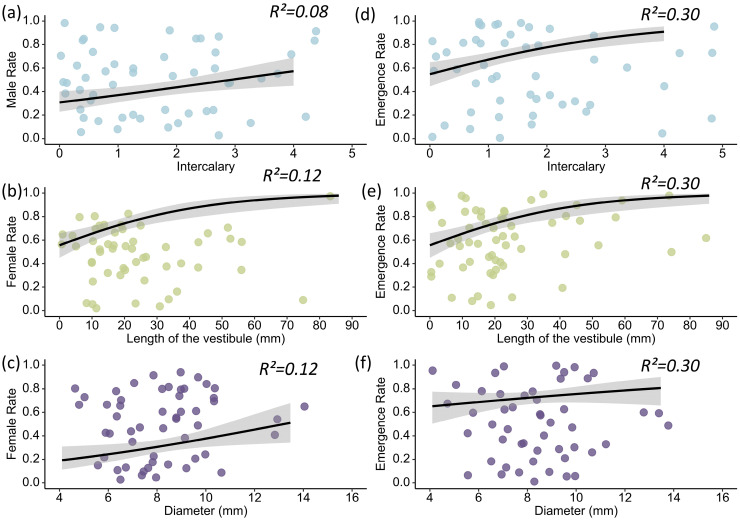
Relationship between the male emergence rate of *D. wangi* and number of intercalary cells (**a**). Relationship between the female emergence rate of *D. wangi* and lengths of nest vestibule (**b**) and nest diameters (**c**). Relationship between the total emergence rate of *D. wangi* and number of intercalary cells (**d**), lengths of nest vestibule (**e**), and nest diameters (**f**). The gray area represents the 95% confidence interval.

In the GLM, the standardized parameter of the number of intercalary cells was significant (Table 1, Figure 4a; Estimate = 0.276 ± 0.124 SE, Z = 2.224, *p* = 0.020), while neither the nest diameter (*p* = 0.787) nor the vestibule length (*p* = 0.467) exhibited a significant relationship.

In the model, the standardized parameter of the nest diameter was significant (Table 1, Figure 4c; Estimate = 0.371 ± 0.143 SE, Z = 2.601, *p* = 0.009), and the vestibule length was marginally significant (Table 1, Figure 4b; Estimate = 0.234 ± 0.142 SE, Z = 1.653, *p* = 0.098). The number of intercalary cells showed no significant relationship (*p* = 0.493). The model accounted for 11.33% (R^2^) of the variation in the female emergence rate. Diameter explained 77.16% of the total variation in the female emergence rate, and vestibule length explained 22.84% (Figure 3b).

In this model, the number of intercalary cells (Table 1, Figure 4d; Estimate = 0.494 ± 0.141 SE, Z = 3.492, *p* = 0.0005), nest cell diameter (Table 1, Figure 4f; Estimate = 0.318 ± 0.144 SE, Z = 2.220, *p* = 0.028), and vestibule length (Table 1, Figure 4e; Estimate = 0.395 ± 0.140 SE, Z = 2.815, *p* = 0.005) all showed significant relationships. The model accounted for 30.23% (R^2^) of the variation in the emergence rate. Diameter explained 14.52% of the total variation in the emergence rate, vestibule length explained 29.77%, and number of intercalary cells explained 55.71% (Figure 3c).

## 4. Discussion

### 4.1. Biological Significance of the Nest-Building Characteristics of Discoelius wangi

This study found that the offspring sex ratios were male-biased, a phenomenon that may be closely related to the species’ reproductive strategy [18]. From an evolutionary perspective, a higher proportion of males can increase the intensity of competition among males during the population breeding period, promoting mating with more dominant males and thereby improving the genetic quality of offspring. This is consistent with the strategy of some Vespidae species to adjust their sex ratio in response to environmental pressures [19]. Males also often disperse more than females, such that a preference for this strategy suggests dispersal is important [20].

In terms of nesting materials and brood cell layout, *D. wangi* uses fragmented leaves to seal nests and construct brood cell walls. This material selection exhibits significant ecological adaptability: fragmented leaves are widely available and easily accessible, which not only effectively block the invasion of external natural enemies but also maintain a relatively stable temperature and humidity environment inside the brood cells, providing a protective barrier for offspring development. Meanwhile, the air permeability of fragmented leaves prevents larval mildew caused by excessive humidity in the brood cells-reflecting an efficient nesting adaptation strategy formed by this species during long-term evolution [21]. These nesting characteristics reflect the resource utilization pattern of *D. wangi* in trap nests: by planning the number of brood cells, the distribution of intercalary cells, and the size of brood cells, this species maximizes reproductive efficiency within the limited space of nest tubes, which also reflects its high adaptability to the habitat environment [8].

### 4.2. Effects of Nest Architecture on the Number of Brood Cells of Discoelius wangi

This study shows that only the number of intercalary cells had a significant positive effect on the total number of brood cells. As a partition structure between brood cells, the number of intercalary cells directly reflects the degree of spatial division within the nest tube. A greater number of intercalary cells means more independent regions are divided within the nest tube, providing more functionally independent units [22]. Furthermore, these interstitial areas may also inhibit easy parasitism or attack, by making the nest appear to be ended [23]. It intuitively makes sense that if intercalary cells are beneficial in these ways, then, as more brood cells are constructed, more intercalary cells will also be built.

In contrast, neither the vestibule length nor the nest diameter had a significant effect on the number of brood cells. This result may be closely related to the functional positioning of the vestibule and nest diameter. The vestibule is a channel structure at the entrance of the brood cells, whose main function is to protect the brood cells from external disturbances. Changes in its length are more likely due to adaptation to the environment of the brood cell entrance or the enhancement of defensive capabilities, rather than directly due to determining the number of brood cells [14]. The nest cell diameter, on the other hand, is related to the spatial requirements for offspring development and needs to be maintained within a range suitable for offspring growth. Excessive changes in the diameter may affect offspring survival [8]. Therefore, *D. wangi* does not increase the number of brood cells by changing the nest diameter; instead, it optimizes the number of brood cells by adjusting the number of intercalary cells within a relatively fixed width diameter range.

### 4.3. Differential Effects of Nest Architecture on the Offspring Quantity of Discoelius wangi

The results revealed that the development of female offspring is highly dependent on the spatial resources of brood cells. Nest diameter determines the amount of food resources that can be accommodated in a single nest cell and the growth space available for offspring; a larger diameter provides more sufficient nutrient reserves and activity space for female larvae, thereby improving the survival rate of female larvae [24]. Meanwhile, the number of intercalary cells also indirectly play a beneficial role. It suggests that female Hymenoptera insects may selectively lay eggs (fertilized or unfertilized) in nests with different parameters by recognizing nest parameters (such as nest diameter) to match the developmental needs of their offspring. For example, they prefer to lay fertilized eggs (females) in nests with larger diameters, utilizing the sufficient space to improve the survival rate of female offspring [25].

Unlike female offspring, the number of intercalary cells is the only factor that exerts a significant positive effect on the number of male offspring, while the effects of nest diameter and vestibule length are negligible. This differential result reflects a significant difference in the developmental space requirements between male and female offspring: male larvae may do well encountering higher variation in nest diameter and can develop normally even in smaller spaces, so nest diameter has a minimal impact on their quantity [10]. In contrast, the number of intercalary cells indirectly determines the reproductive quantity of male offspring by determining the total number of brood cells (through intercalary cell benefits), making it the only key factor affecting their quantity [8].

Notably, we recognize that the sex ratio of emerging adults may be influenced by factors beyond nest architecture. First, a density-dependent sex allocation strategy, commonly observed in many insects, could be at play: females may adjust offspring sex ratio in response to brood cell density, investing in fewer, high-quality (female) offspring or more, smaller (male) offspring, a strategy often linked to Local Mate Competition. Second, differential food resource allocation constitutes another critical factor. Female wasps might provision larger lepidopteran larvae to female-destined brood cells and smaller prey items to male-destined cells, which directly affects offspring survival and final sex ratios independently of nest structure [26].

### 4.4. The Complex Regulation of Nest Architecture on the Emergence Rate of Offspring in D. wangi

Our results suggest that female larvae have stricter requirements for brood cells, especially in terms of space. Generally, female vespidae wasps are larger in size than males, and their development requires more food and space [6,27]. In smaller brood cells, nutritional resources may be more limited, making it difficult for larvae to develop adequately for reproduction; in contrast, a larger nest diameter can better meet the needs of female offspring. A high concentration of energy and nutrient supply is beneficial for solitary wasps and bees, thereby improving the emergence success rate [28].

The female emergence rate is directly related to the reproductive potential of the population. As a core factor affecting the female emergence rate, the regulatory mechanism of nest diameter reflects the high priority that *D. wangi* places on the survival of female offspring [29]. During the long-term evolutionary process, this species may have created optimal spatial conditions for the emergence of female offspring by selecting appropriate nest diameter (with a nest diameter range of 4.10 mm–13.47 mm in this study). This is a key adaptive strategy for the species to ensure reproductive performance and maintain population continuation [30]. It seems likely that they balance these concerns with the need for males simply by using smaller nest cell spaces for males while prioritizing larger potential cell areas for females.

This result suggests that the number of intercalary cells may indirectly regulate the male emergence rate by influencing the independence between brood cells. The presence of intercalary cells creates physical partitions between brood cells. A greater total number of these partitions within a nest tube provides more distinct spatial separation among the brood cells, which can reduce interference (e.g., food competition, pest and disease transmission) among larvae from different brood cells during development and emergence, as well as reduce attacks, thereby providing a more stable environment for the emergence of male larvae [31,32]. Unlike females, male larvae have stronger adaptability to nest diameter, so nest diameter has a smaller impact on their emergence rate [10]. Overall, the number of intercalary cells is a potential key factor affecting the male emergence rate; its mechanism of action may be related to ensuring the independence of brood cells and reducing interference. Sufficient space can effectively reduce the risk of larval damage caused by extrusion or collision [33].

This result reveals the synergistic effect of nest diameter, vestibule length, and the number of intercalary cells in ensuring overall emergence success. As the “first line of defense” for brood cells, a longer vestibule provides a stronger protective effect on the internal environment of brood cells: it can effectively block the invasion of external natural enemies (e.g., parasitoid wasps, ants) and the impact of external environmental fluctuations (e.g., changes in temperature and humidity), thereby providing a stable external environment for the emergence of all offspring [34]. Meanwhile, the number of intercalary cells enhances the independence of brood cells, reduces internal interference, and further improves the overall emergence success rate [31,32].

The overall emergence rate is a comprehensive indicator for measuring population reproductive performance. The synergistic regulation of vestibule length and the number of intercalary cells improve the overall successful emergence rate, which reflects *D. wangi*’s strategy of ensuring offspring survival from two dimensions: “external defense” and “internal optimization” [7].

## 5. Conclusions

In conclusion, *Discoelius wangi* achieves precise regulation of reproductive performance by differentially adjusting nest architecture parameters, with the number of intercalary cells playing a dominant role in spatial distribution and male production. By modifying the number of intercalary cells, the species realizes efficient resource allocation—flexibly regulating the number of male offspring without increasing nest diameter, while concentrating resources to improve the developmental quality of female offspring—thus ensuring population persistence. This strategy reflects a refined adaptive mechanism formed during the species’ long-term evolution. This study identifies the key structural elements underlying the reproductive adaptation of *D. wangi*, providing a theoretical basis for the artificial breeding (e.g., optimizing intercalary cells design) and ecological conservation (e.g., maintaining plant diversity for nesting materials) of vespid species.

## Figures and Tables

**Table 1:** Summary of Generalized Linear Model Results for the Number of Males and Females and the Number of Brood cells, and the Emergence Rate.

Male	Estimate	Std. Error	Z-Value	*p*
(Intercept)	0.163	0.112	1.449	0.147
Intercalary	0.291	0.114	2.552	0.011
**Female**	**Estimate**	**Std. Error**	**Z-Value**	***p***
(Intercept)	−0.128	0.119	−1.072	0.274
Diameter	0.275	0.116	2.373	0.018
Intercalary	0.233	0.117	1.999	0.046
**Brood Cells**	**Estimate**	**Std. Error**	**Z-Value**	***p***
(Intercept)	1.071	0.060	17.937	<0.001
Intercalary	0.141	0.061	2.324	0.020
**Male Rate**	**Estimate**	**Std. Error**	**Z-Value**	***p***
(Intercept)	−0.386	0.124	−3.121	0.002
Intercalary	0.276	0.124	2.224	0.026
**Female Rate**	**Estimate**	**Std. Error**	**Z-Value**	***p***
(Intercept)	−0.804	0.132	−6.105	<0.001
Diameter	0.371	0.143	2.601	0.009
Length of the vestibule	0.234	0.142	1.653	0.098
**Emergence Rate**	**Estimate**	**Std. Error**	**Z-Value**	***p***
(Intercept)	1.007	0.142	7.072	<0.001
Diameter	0.318	0.144	2.200	0.028
Length of the vestibule	0.395	0.140	2.815	0.005
Intercalary	0.494	0.141	3.492	0.0005

## Data Availability

The original contributions presented in this study are included in the article/Supplementary Materials. Further inquiries can be directed to the corresponding authors.

## Supplementary Materials

The following supporting information can be downloaded at: https://www.mdpi.com/article/10.3390/insects16121197/s1, Table S1: Summary of Generalized Linear Model Results for the Number of Males and Females and the Number of Brood cells, and the Emergence Rate; Figure S1. Relationship between the male number of *D. wangi* and length of the vestibule (a) and nest diameters (b). Relationship between the female number of D. wangi and length of the vestibule (c). Relationship between the brood cells of *D. wangi* and nest diameters (d) and length of the vestibule (e). Relationship between the male emergence rate of *D. wangi* and nest diameters (f) and length of the vestibule (g). Relationship between the female emergence rate of *D. wangi* and number of intercalary cells (h); Figure S2. Analysis of the relative contributions of nest cell diameter, intercalary cells and vestibule length to the number of males (a), the number of females (b), and the number of brood cells (c), and the male emergence rate (d), the female emergence rate (e) and the emergence rate (f) of *Discoelius wangi*.
