# Supplementary material for: Nest Architecture Drives Sex-Specific Emergence Success in a Predator Wasp (Hymenoptera, Vespidae, Discoelius wangi)
Source: Insects. 2025 Nov 25;16(12):1197. doi: 10.3390/insects16121197 (PMC12733433; doi:10.3390/insects16121197)
Supplement: Supplementary file 1 [file insects-16-01197-s001.zip › insects-3960468-supplementary.pdf]

Electronic Supplementary Information Appendix 1

Table S1. Summary of Generalized Linear Model Results for the Number of Males and Females and the Number of Brood cells, and the Emergence Rate

Male	Estimate	Std. Error	Z-value	*P*
(Intercept)	0.163	0.112	1.449	0.148
Diameter	0.017	0.116	0.150	0.881
Length of the vestibule	0.006	0.123	0.047	0.962
Intercalary	0.290	0.115	2.518	0.012
Female	Estimate	Std. Error	Z-value	*P*
(Intercept)	-0.130	0.119	-1.094	0.274
Diameter	0.295	0.123	2.419	0.016
Length of the vestibule	0.078	0.125	0.621	0.534
Intercalary	0.221	0.118	1.875	0.061
Brood cells	Estimate	Std. Error	Z-value	*P*
(Intercept)	1.071	0.060	17.809	<0.001
Diameter	0.038	0.063	0.598	0.550
Length of the vestibule	0.068	0.063	-1.066	0.287
Intercalary	0.149	0.061	2.424	0.015
Male rate	Estimate	Std. Error	Z-value	*P*

(Intercept)	-0.379	0.124	-3.056	0.002
Diameter	-0.342	0.127	-0.270	0.787
Length of the vestibule	0.0974	0.134	0.727	0.467
Intercalary	0.270	0.126	2.146	0.032
Female rate	Estimate	Std. Error	Z-value	*P*
(Intercept)	-0.817	0.134	-6.121	<0.001
Diameter	0.362	0.143	2.523	0.012
Length of the vestibule	0.223	0.144	1.550	0.121
Intercalary	0.090	0.131	0.685	0.493
Emergence rate	Estimate	Std. Error	Z-value	*P*
(Intercept)	1.007	0.142	7.072	<0.001
Diameter	0.318	0.144	2.200	0.028
Length of the vestibule	0.396	0.140	2.815	0.005
Intercalary	0.494	0.141	3.492	0.0005

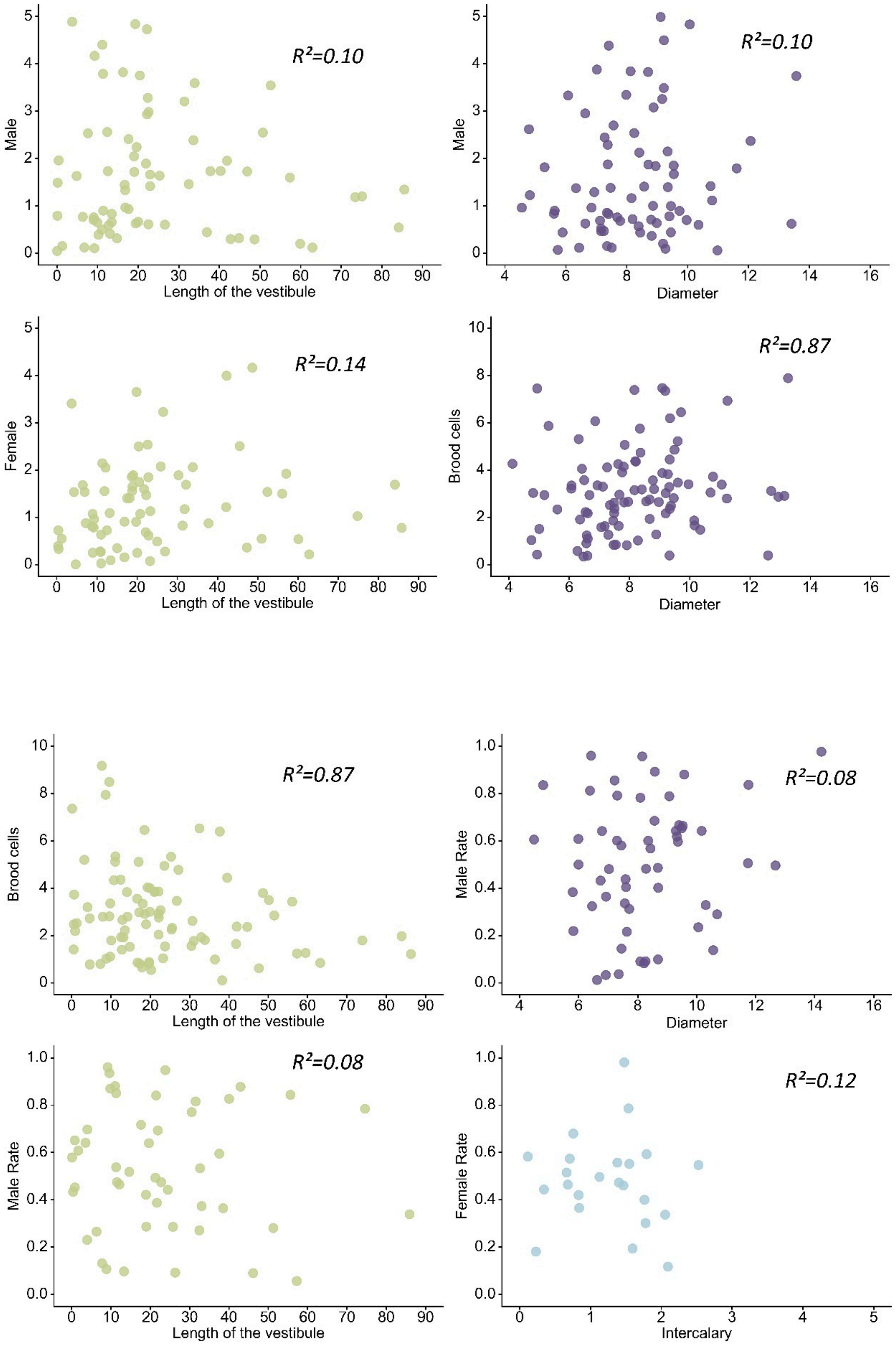

Figure S1. Relationship between the male number of *D. wangi* and length

of the vestibule (a), and nest diameters (b). Relationship between the female number of *D. wangi* and length of the vestibule (c). Rela-tionship between the brood cells of *D. wangi* and nest diameters (d) and length of the vestibule (e). Relationship between the male emergence rate of *D. wangi* and nest diameters (f), and length of the vestibule (g). Relationship between the female emergence rate of *D. wangi* and number of intercalary cells (h). The gray area represents the 95% confidence interval.

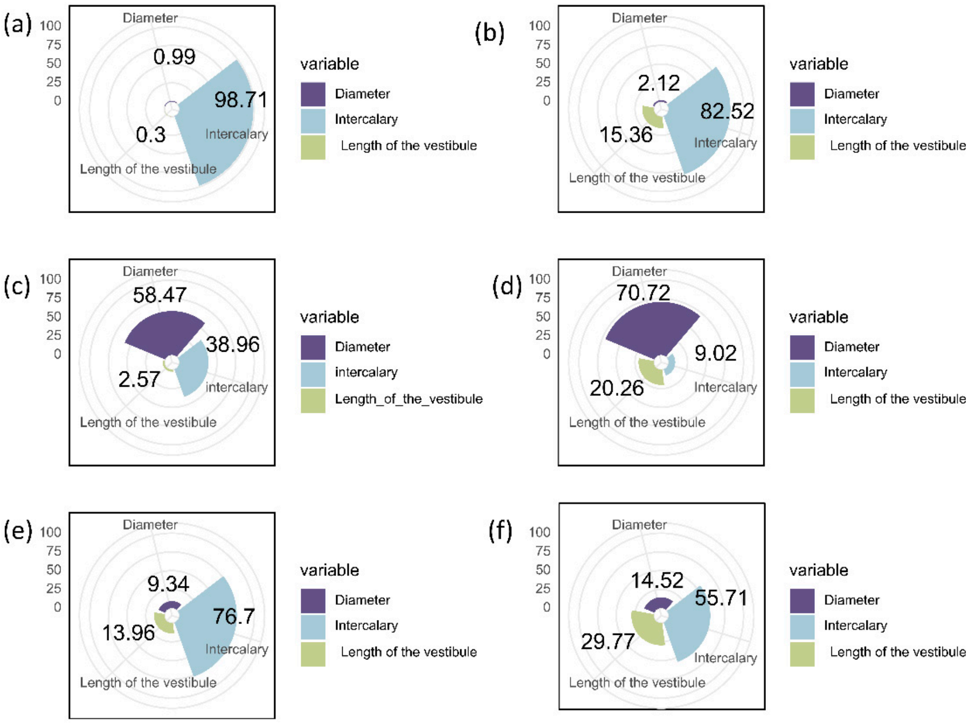

Figure S2. Analysis of the relative contributions of nest cell diameter, intercalary cells and vestibule length to the number of males (a), the number of females (b), and the number of brood cells (c), and the male emergence rate (d), the female emergence rate (e) and the emergence rate (f) of *Discoelius wangi*.
